# Exploring potential biomarkers and therapeutic targets in inflammatory bowel disease: insights from a mega-analysis approach

**DOI:** 10.3389/fimmu.2024.1353402

**Published:** 2024-03-06

**Authors:** Edia Stemmer, Tamar Zahavi, Maoz Kellerman, Liat Anabel Sinberger, Guy Shrem, Mali Salmon‐Divon

**Affiliations:** ^1^ Department of Molecular Biology, Ariel University, Ariel, Israel; ^2^ Kaleidoo, Bar Lev High Tech Park, Misgav, Israel; ^3^ Obstetrics, Gynecology and Infertility (OB&GYN) Department Maccabi Healthcare Services, Tel Aviv, Israel; ^4^ Adelson School of Medicine, Ariel University, Ariel, Israel

**Keywords:** inflammatory bowel disease, ulcerative colitis, Crohn’s disease, biomarkers, mega-analysis, machine learning

## Abstract

**Background:**

Understanding the molecular pathogenesis of inflammatory bowel disease (IBD) has led to the discovery of new therapeutic targets that are more specific and effective. Our aim was to explore the molecular pathways and genes involved in IBD pathogenesis and to identify new therapeutic targets and novel biomarkers that can aid in the diagnosis of the disease.

**Methods:**

To obtain the largest possible number of samples and analyze them comprehensively, we used a mega-analysis approach. This involved reprocessing raw data from multiple studies and analyzing them using bioinformatic and machine learning techniques.

**Results:**

We analyzed a total of 697 intestinal biopsies of Ulcerative Colitis (n = 386), Crohn’s disease (n = 183) and non-IBD controls (n = 128). A machine learning analysis detected 34 genes whose collective expression effectively distinguishes inflamed biopsies of IBD patients from non-IBD control samples. Most of these genes were upregulated in IBD. Notably, among these genes, three novel lncRNAs have emerged as potential contributors to IBD development: *ENSG00000285744*, *ENSG00000287626*, and *MIR4435-2HG*. Furthermore, by examining the expression of 29 genes, among the 34, in blood samples from IBD patients, we detected a significant upregulation of 12 genes (p-value < 0.01), underscoring their potential utility as non-invasive diagnostic biomarkers. Finally, by utilizing the CMap library, we discovered potential compounds that should be explored in future studies for their therapeutic efficacy in IBD treatment.

**Conclusion:**

Our findings contribute to the understanding of IBD pathogenesis, suggest novel biomarkers for IBD diagnosis and offer new prospects for therapeutic intervention.

## Introduction

Ulcerative colitis (UC) and Crohn’s disease (CD), both forms of inflammatory bowel disease (IBD), are chronic immune-mediated inflammatory diseases that affect the digestive system, characterized by periodic episodes of relapse and remission and currently lacking a definitive cure (1, 2). While UC mainly targets the large intestine (colon), causing continuous surface inflammation, starting from the rectum and extends along the colon, CD can affect any part of the digestive tract, often exhibiting a patchy pattern and frequently occurring in the small intestine, particularly the terminal ileum (3). However, distinguishing between CD and UC in some cases can be challenging, leading to an interim diagnosis of “indeterminate” or “unclassified” colitis and potential treatment delays. Currently, indeterminate colitis remains unresolved in 5-15% of IBD patients (4).

The management of IBD has undergone significant transformation in the past two decades, thanks to the emergence of biological therapies. However, not all patients respond to these biological drugs, and the prevalence of IBD is on the rise globally (5). Hence, early diagnosis and prompt treatment initiation are pivotal strategies to enhance patient outcomes and overall well-being (6).

Upon initiating effective therapy, confirming remission using measurable endpoints is crucial. objective endpoints such as endoscopic, histological, and clinical measures, alongside surrogate biomarkers like blood CRP or fecal calprotectin levels are helping to evaluate and understand the state of the disease. However, the practical application of endoscopic and fecal measurements poses challenges in routine practice and lacks specificity for intestinal inflammation (7). Previous study has emphasized the development of a new molecular measurement, focusing on molecular signature of mucosal and peripheral blood components to enhance accuracy and predict relapse (8).

Recently, the understanding of the molecular pathogenesis of IBD has advanced significantly leading to enhanced disease management. Importantly, specific signaling pathways have emerged as having a pivotal role in the inflammatory process, contributing to dysregulated inflammatory responses and playing essential roles in the development of IBD (9). Key pathways involved in this process include NF-κB pathway, PI3K/Akt signaling pathway, and JAK/STAT signaling pathway (10). Additionally, MAPK signaling pathway, Chemokine signaling pathway, Cytokine-cytokine receptor interaction pathway are significant contributors to diseases associated with inflammation including IBD (11–13). A deeper investigation into these biological pathways may reveal new targets for therapeutic interventions and facilitate the discovering of diagnostic and monitoring biomarkers.

Over the years, extensive research has been conducted on transcriptomic data and gene signatures associated with IBD. The introduction of next-generation sequencing (NGS) has greatly improved our ability to study the disease by providing higher resolution, increased sensitivity, and the capacity to discover novel IBD-related molecular transcripts. Despite these advances, integrating findings from different studies has been challenging due to experimental variations and varying analytical methods. To overcome these challenges, we adopted a comprehensive mega-analysis approach that involved reprocessing and consolidating data from multiple sources. Through this large-scale analysis and the application of standardized methods across the combined dataset, we identified novel biomarkers for IBD diagnosis and offer new prospects for therapeutic intervention.

## Materials and methods

**Figure 1:**
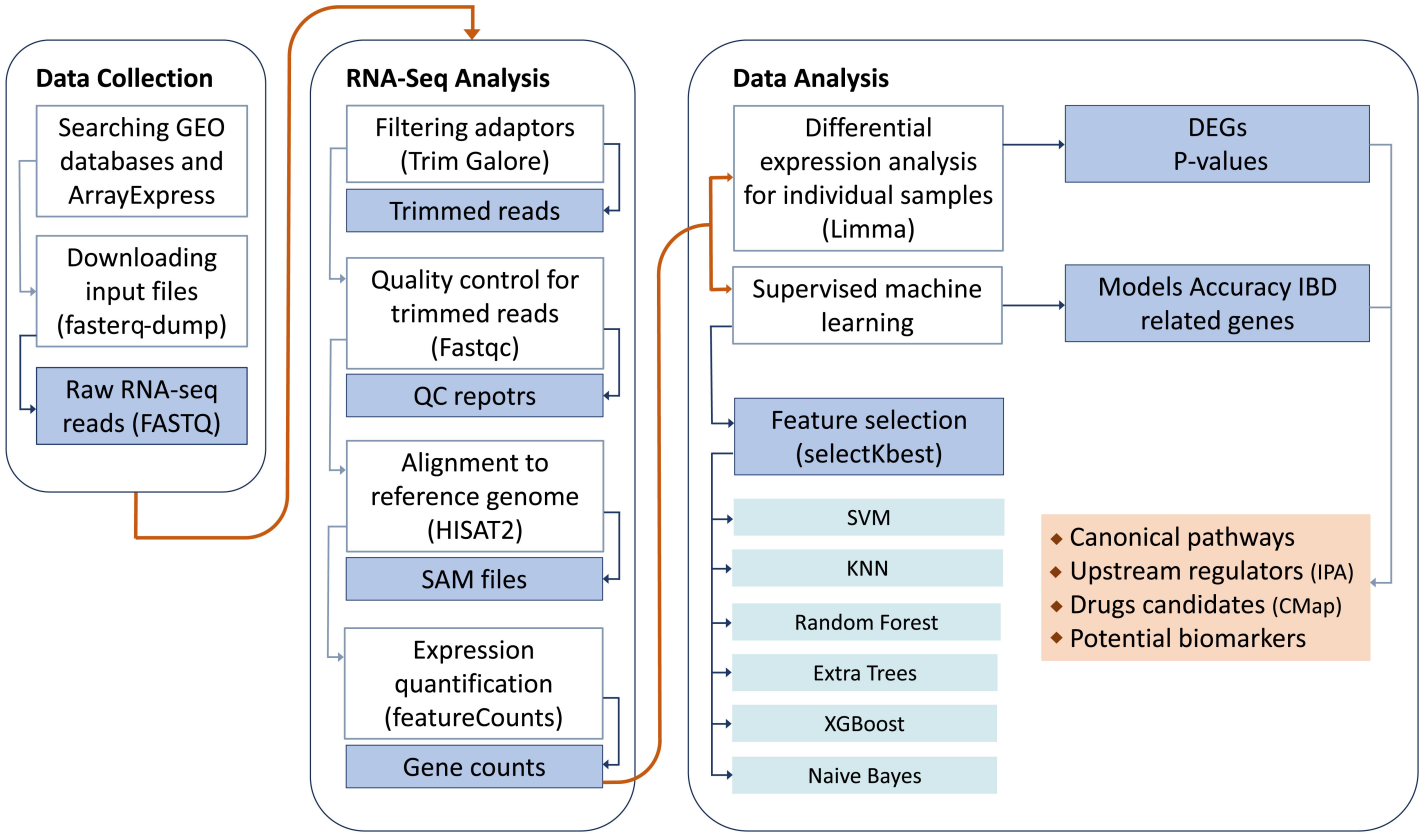
Study design.

To perform the mega-analysis, we conducted a thorough search in public databases for relevant studies. The datasets, which contained raw data, were then downloaded, and consistently reprocessed to facilitate subsequent analyses. The entire study design is visually depicted in **Figure 1**.

### Search strategy and study selection criteria

**Figure 2:**
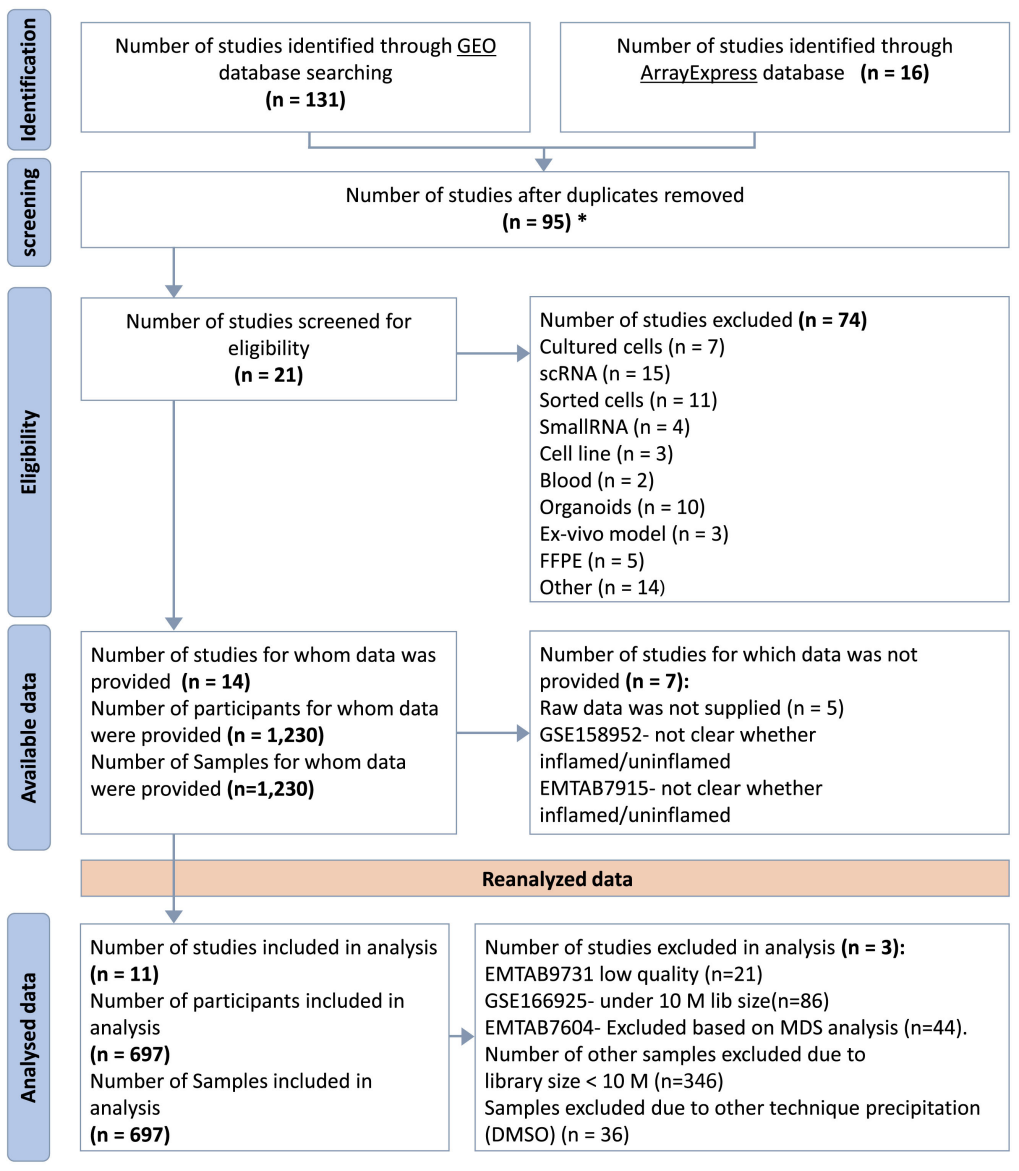
PRISMA Flow Diagram. *Duplicated samples have been removed. GSE62207, GSE93624 - used same patients taken from RISK project, duplicated with GSE101794. GSE150961- used same patients as in GSE109142 taken from the PROTECT.

Comprehensive searches of Gene Expression Omnibus (GEO) (14) and ArrayEpress (AE) (15) databases were performed on October 5, 2021. The search terms were as follows: (“Inflammatory Bowel Disease”) OR (“Crohn’s disease”) OR (“Ulcerative colitis”) AND (“high throughput sequencing”[Platform Technology Type]) AND “homo sapiens”[Organism] AND (“RNA-seq” OR “RNAseq” OR “RNA sequencing”). This systematic review was performed according to the Preferred Reporting Items for Systematic Reviews and Meta-Analyses (PRISMA) guidelines (16). The flow chart for the selection of studies is shown in **Figure 2**.

### Inclusion/exclusion criteria

Only studies meeting specific inclusion criteria were considered for the mega-analysis. These criteria included the availability of RNA-seq raw data, transcriptomes obtained from whole tissue samples (punch biopsy), direct sequencing without any further manipulations (e.g. tissue culture or isolated cells), the use of the illumina sequencing platform, and the availability of metadata. Studies based on microarray platform, single-cell RNA-seq and small RNA-seq were excluded from the analysis. Additionally, uninflamed biopsies were excluded due to the limited size of the retrieved cohort.

### RNA sequencing data processing

We uniformly processed the RNA sequencing data from the selected eligible studies. Raw data were downloaded using the SRA-Toolkit (https://hpc.nih.gov/apps/sratoolkit.html). Briefly, we utilized the fasterq-dump tool to download FASTQ files, followed by adapter trimming using Trim Galore (https://www.bioinformatics.babraham.ac.uk/projects/trim_galore/) and quality checking with FASTQC (https://www.bioinformatics.babraham.ac.uk/projects/fastqc/). Next, we aligned the high-quality reads to the reference genome (GRCh38) using HISAT2 (17). The number of reads mapped to each annotated genes was then counted using featureCounts software (18). For the statistical analysis we retained only samples with a library size greater than 10 million counts, and genes with less than 1 count per million reads (CPM) across more than 90 samples were filtered out using the ‘filterByExpr’ function implemented in edgR package (19).

### Differential expression analysis

Normalization and differential gene expression were conducted using the edgeR (19) and limma (20) R packages. In brief, raw read counts were normalized using the TMM method and then a voom transformation was applied to approximate a normal distribution, resulted in a dataset with logCPM values. Prior to initiating the differential expression analysis, we performed Multidimensional Scaling (MDS) analysis to represent the data in a reduced-dimensional space which enable the visualization of batch effects associated with the dataset. We found the “study source” factor to have the largest contribution to the variation, with a notable correlation to the tissue source (small intestine vs. large intestine) (**Supplementary Figure 1**). Following the consideration of study batch effects, we applied linear modelling to detect differential expression. Statistically significant differentially expressed genes (DEGs) were defined as those having fold change > 2 and False Discovery Rate (FDR) < 0.05.

To distinguish between UC and CD, we utilized samples from the large and small intestines, specifically from the Rectum (UC= 233, non-IBD control= 46) and from the Ileum (CD= 155, non-IBD control= 24), respectively. We added an interaction term to the linear model, in order to detect which genes respond differently to the disease in UC compared to CD. To model the tissue effect we added a comparison between the small intestine and the large intestine among the non-IBD control samples. The study feature was added as covariate to the model to account for its effect.

Linear equation used to distinguish between each disease type and control samples as well as analysis details are described in **Supplementary Materials** under methods section.

### Pathway and upstream regulators analysis

To identify canonical pathways and upstream regulators, we used the Ingenuity Pathway Analysis (IPA) (21) based on list of DEGs. Briefly, we performed the core analysis to identify significant canonical pathways that were enriched among the DEGs and to predict significant activated upstream regulators. Negative z score value implies an overall pathway’s inhibition and a positive z-score value suggests an overall pathway’s activation. Pathways and upstream regulators having p-value < 0.05 and absolute z‐score ≥ 2 were considered statistically significant.

### Machine learning

We employed supervised machine learning (ML) techniques on the gene expression data for classifying various diagnoses: UC (n= 233, Rectum) vs. non-IBD control (n= 46, Rectum), CD (n= 164, Small intestine) vs. non-IBD control (n= 33, Small intestine), and IBD (388 cases, including 233 UC rectum and 155 CD ileum) vs. non-IBD control (70 samples, including 46 rectum and 24 ileum). These diagnoses were treated as the output variables. Each diagnosis was subjected to separate analysis using various ML algorithms to develop a predictive model for classification.

To ensure an unbiased evaluation of the model’s performance, we split our data into training (80%) and test (20%) sets using the train_test_split function from the Scikit-learn (22) library in Python (23). After applying feature selection, we trained and tested different classifier models, followed by validation using the GSE193677 dataset as an external and independent data source (as detailed below).

1.*Feature selection*

To reduce the number of features and select input variables that have the strongest relationship with the output variables we utilized the SelectKBest algorithm from the Scikit-learn library. This algorithm ranks the features based on their individual ANOVA F-values and selects the top k features with the highest F-values as the most relevant for prediction. This approach is suitable for categorical output variables, as in our study. Evaluation of the selected features was performed using a Logistic Regression classifier with different random states and cross-validation. This allowed us to assess the effectiveness of the selected k features in accurately predicting the target variable. By iterating through different k values and random states, we were able to identify the combination that yielded the highest accuracy, indicating the optimal number and significance of features. The k values and random state are described in **Supplementary Table 1**.

2.*Classifiers*

In our analysis, we utilized six classifier models from the Scikit-learn (22) and xgboost (24) packages: SVM, KNN, Random Forest, Extra Trees, XGBoost and Naive Bayes. To optimize their performance, we implemented a nested loop to iterate over different hyperparameter values for each algorithm. These hyperparameters were tuned to enhance predictive capabilities and mitigate overfitting for each model (**Supplementary Table 1**).

To distinguish between UC and CD, we first run DE analysis as described above then, we selected genes meeting two criteria: 1) significant interaction (fold change > 2 and FDR < 0.05) and 2) non-significance in tissue comparison (defined as fold change < 1.3 and FDR > 0.5). This resulted in 365 genes. These genes were subsequently used as features in a ML analysis. The expression matrix used as input to ML analysis contained normalized expression values that were corrected for tissue and study batch effect using removeBatchEffect() function from the limma package (20). The ML analysis, included 233 UC-inflamed Rectum samples and 155 CD-inflamed Ileum samples for training the models. For validation, we utilized data from the GSE193677 dataset, which included 60 UC-inflamed Rectum samples, 68 CD-inflamed Rectum samples and 60 CD inflamed ileum. Feature selection and ML analysis was done as described above.

### Independent validation data

As an independent validation dataset we used the transcriptomic data from the Argmann et al. study (8), available on GEO database (GSE193677). The raw data was downloaded and underwent reprocessing steps, including alignment to the GRCh38 reference genome and voom transformation. The dataset downloaded consisted of 240 samples. For our analysis, we included 60 samples from patients diagnosed with UC, showing severe to moderate inflammation, collected from the rectum, and 60 samples from patients with CD, exhibiting similar inflammation levels, obtained from the ileum. Additionally, we incorporated 120 samples from non-IBD control participants, 60 samples from the rectum and 60 samples from the ileum. We maintained an equal representation of male and female participants in all groups. The dataset was used to validate the six different classifier models. The accuracy and area under the ROC curve (AUC) were calculated to assess models’ performance.

### Blood samples dataset

The blood samples dataset was obtained from the Argmann et al. study (8) available on GEO database (GSE186507) which comprises blood RNA-seq data collected from whole blood samples during the participant’s endoscopy visits, along with comprehensive clinical, and endoscopic evaluations. We downloaded and reanalyzed (as described above) a total of 111 samples collected from patients diagnosed with both UC and CD. These samples were from individuals exhibiting clinical symptoms along with either severe or inactive endoscopic states. Additionally, we included samples from patients without clinical symptoms and with an inactive endoscopic state, as well as samples from non-IBD controls.

### Drug repurposing analysis

The drug repurposing analysis was conducted using the Connectivity Map (CMap) (25) through the web application CLUE (https://clue.io) (26). CMap is a collection of genome-wide transcriptional expression data responses from human cell lines that have been treated with chemical compounds known as perturbagens. These perturbagens induce changes in cellular gene expression patterns. Our aim was to identify perturbagens that induced opposing expression patterns, to the top significant 300 upregulated and downregulated genes within the IBD vs. non-IBD control DEGs, and within the comparison between each of the diseases and non-IBD control samples separately. Querying these expression patterns with the CMap reference, particularly the Gene Expression (L1000) Touchstone dataset, generated a heatmap displaying the connectivity score (tau) of 2837 small-molecule compounds as perturbagens. Our focus was on compounds demonstrating the highest anti-correlation and CMap median tau score below −95 in the HT-29 cell line column. These compounds are candidates for drug repurposing. We chose to look at HT-29 cells, since this cell line, originating from colon adenocarcinoma, serves as a relevant *in-vitro* model for studying processes like absorption, transport, and secretion by intestinal cells (27–29).

### Statistical analysis

Statistical analyses were conducted using the R statistical framework (v.4.2.2) (30). Boxplots illustrating expression levels were generated using the ggplot2 R package (v.3.4.0) (31). Unless indicated otherwise, comparisons between different groups were calculated using t test or ANOVA, with a significance threshold set at p < 0.05. The statistical analysis involved training and evaluating six machine learning algorithms was done using Python (v.3.10.11) (23). Model performance was assessed using the accuracy_score function to compute classification accuracy, while Receiver Operating Characteristic (ROC) curves were utilized to evaluate the model’s ability to distinguish between classes, with the AUC (Area Under the Curve) serving as a measure of classifier performance. The scikit-learn (v.1.2.0) (22) library was employed for computation, and matplotlib (v.3.6.2) (32) for visualization.

## Results

### Datasets and cohorts analyzed in the study

Based on our inclusion criteria, 11 datasets were selected and subsequently were included in the mega-analysis. The datasets included clinical data and biopsy samples from the intestines of a total of 697 participants, comprising 569 IBD patients and 128 non-IBD controls (**Figure 2**). Distribution of expression data pre and post normalization is given in (**Supplementary Figure 2**).

The 11 eligible studies included in the analysis are described in **Table 1**. Within our UC cohort, which is derived from eight of these studies, we obtained a total of 386 inflamed biopsies specifically from the large intestine. For the CD cohort, which is derived from four studies, we identified 183 inflamed biopsies primarily from the small intestine. Among the included studies, five provided non-IBD control samples, mostly from subjects suspected of having IBD but with radiographic, endoscopic, and histologic findings. The remaining samples obtained from normal bowel sections, located more than 10 cm away from tumors in patients undergoing bowel resection for sporadic colon cancer. Overall, the control group included 95 samples from the large intestine and 33 samples from the small intestine.

**Table 1:** Characteristic of datasets used for analysis.

Study No.	Dataset	Small/Large Intestine	Sample Size	CD	UC	Control
1	GSE101794	Small	157	135	–	22
2	GSE137344	Small	1	1	–	–
3	GSE83687	Small/Large	123	28/11	-/27	11/46
4	GSE107593	Large	12	–	12	–
5	GSE108746	Large	18	–	18	–
6	GSE109142	Large	226	–	206	20
7	GSE117993	Large	41	–	16	25
8	GSE130038	Large	25	–	25	–
9	GSE66207	Large	4	–	–	4
10	GSE72819	Large	66	–	66	–
11	EMTAB7845	Large	24	8	16	–
**Total**			**697**	**183**	**386**	**128**

### Inflammatory pathways and upstream regulators common between UC and CD

**Figure 3:**
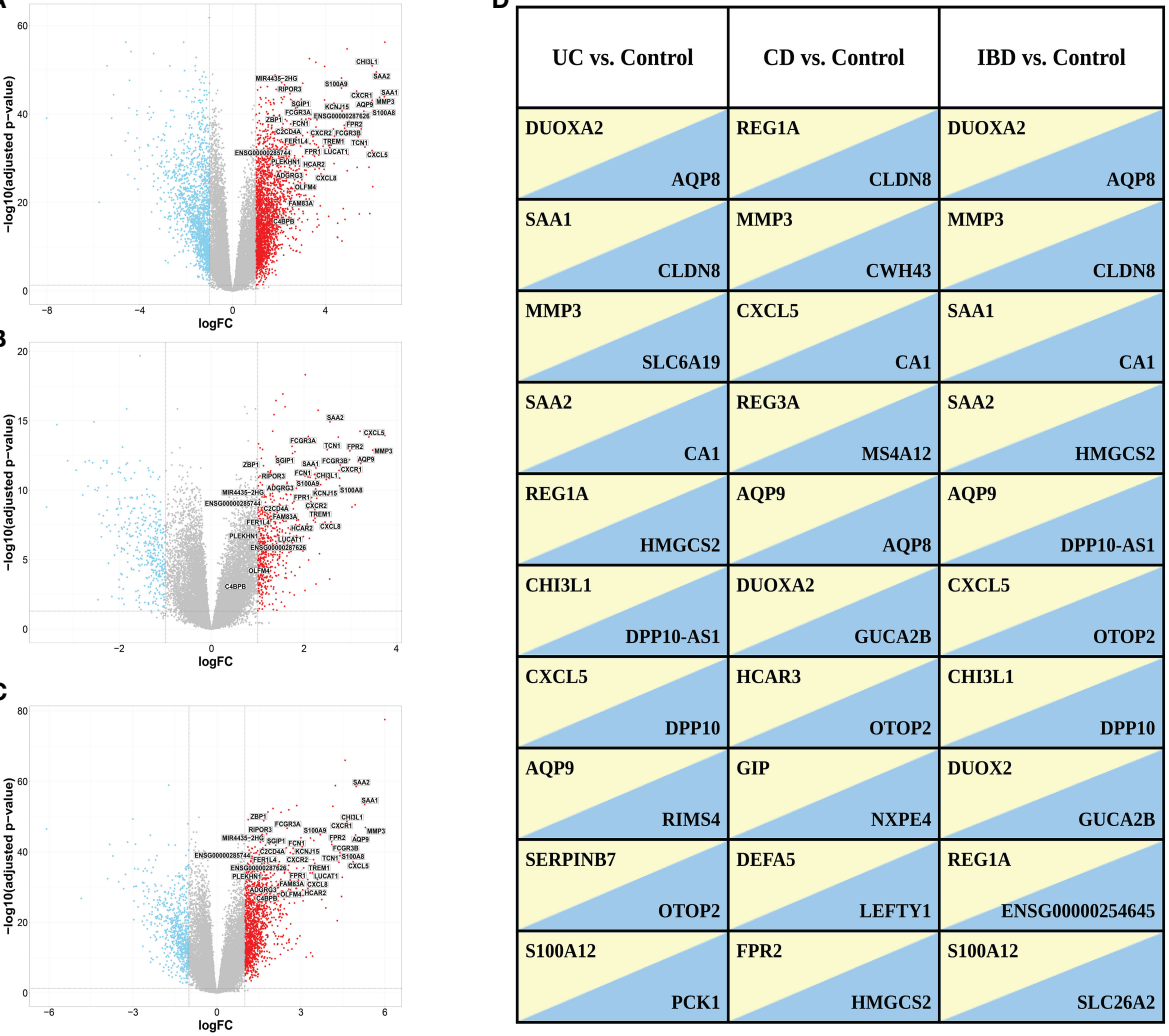
Volcano plots of DEGs selected with a threshold of adjusted p-value < 0.05 and fold-change >2 for the following comparisons: **(A)** UC vs. control, **(B)** CD vs. control and **(C)** IBD vs. control. Highlighted are the 34 genes found in ML analysis (see ML analysis section below). **(D)** Top ten up (*yellow*)- and down (*blue*)-regulated genes in the different comparisons. Genes with FDR < 0.05 were ranked based on fold change.

We identified 4,027 DEGs (2396 upregulated and 1631 downregulated genes, **Figure 3A**) when comparing gene expression from inflamed biopsies of the UC group and the control group, both taken from the large intestine. Given the involvement of CD in both the small and large intestines, we merged data of the biopsies obtained from inflamed regions of both intestinal segments. We then compared this combined dataset to non-IBD control biopsies collected also from both the small and large intestines. By conducting this comparative analysis, we sought to gain insights into the gene expression patterns associated with CD across both tissues. We found 1,008 DEGs including 629 upregulated genes and 379 downregulated genes in inflamed samples vs. non-IBD controls (**Figure 3B**). Finally, we chose to investigate IBD as a unified group when compared to the control group. In the IBD vs. control group we identified 2099 DEGs including 1387 upregulated and 712 downregulated. (**Figure 3C**). The top ten up- and down-regulated genes are displayed in **Figure 3D**. Several genes consistently appear across all comparisons. Notably, a lncRNA *ENSG00000254645*, stands out as it ranks among the top ten downregulated genes in the IBD vs. Control comparison. This transcript has previously been associated with IBD (33). In our analysis, this transcript demonstrates significant downregulation in both the CD vs. Control and UC vs. Control comparisons (**Supplementary Figure 3**).

**Figure 4:**
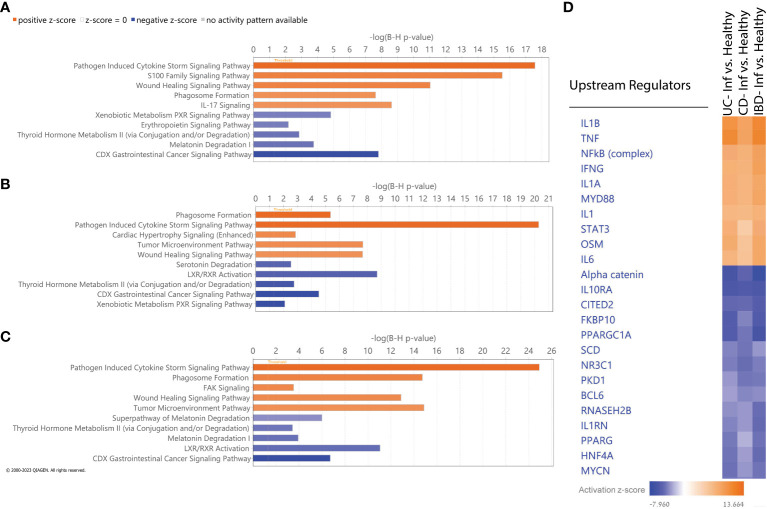
IPA Functional analysis. **(A-C)** Top five activated (orange) and inhibited (blue) canonical pathways based on absolute IPA’s activation z-score > 2 for the following comparisons: **(A)** UC vs non-IBD control, **(B)** CD vs non-IBD control, **(C)** IBD vs non-IBD control. Negative z score value implies an overall pathway's inhibition and a positive z-score value suggests an overall pathway's activation. All pathways had -log (B-H p-value) > 1.3 meaning FDR less than 0.05. **(D)** Hierarchical clustering of upstream regulators’ activation z score across all comparisons.

We utilized IPA to identify molecular pathways enriched within the list of DEGs obtained by comparing inflamed to non-IBD control tissues in the different comparisons (**Figures 4A–C**). As expected, activated pathways in all comparisons, were enriched in immune response pathways, including pathogen induced cytokine storm signaling, phagosome formation pathway and wound healing signaling. IL-17 was also upregulated in all comparisons, although did not rank in the top five upregulated pathways in CD and IBD comparisons. The downregulated pathways observed in all comparisons encompassed various metabolism and catabolism processes. For example; serotonin degradation, nicotine degradation III, thyroid hormone metabolism II (via conjugation and/or degradation), and melatonin degradation I. Along with the metabolism pathways there are also downregulated nuclear receptor (NR) pathways which related directly to gut microbiota and play protective roles in intestinal epithelial integrity (34). Notably, LXR/RXR Activation signaling, which is a NR pathway, was downregulated only in CD and IBD comparisons, but not in UC. IPA Comparison analyses revealed activated and inhibited upstream regulators (**Figure 4D**). Upon the common activated regulators we found pro-inflammatory cytokines (IL1B, TNF), and the NFkB complex which trigger immune responses. Upon the common inhibited regulators we identified alpha catenin and the IL10RA which their inhibition is known to increase intestinal permeability (35) and promotes inflammation (36), respectively. Overall, these results show that similar pathways and upstream-regulators are involved in both diseases and suggest that similar molecular mechanisms operate in both.

### Machine learning prediction models for IBD

**Figure 5:**
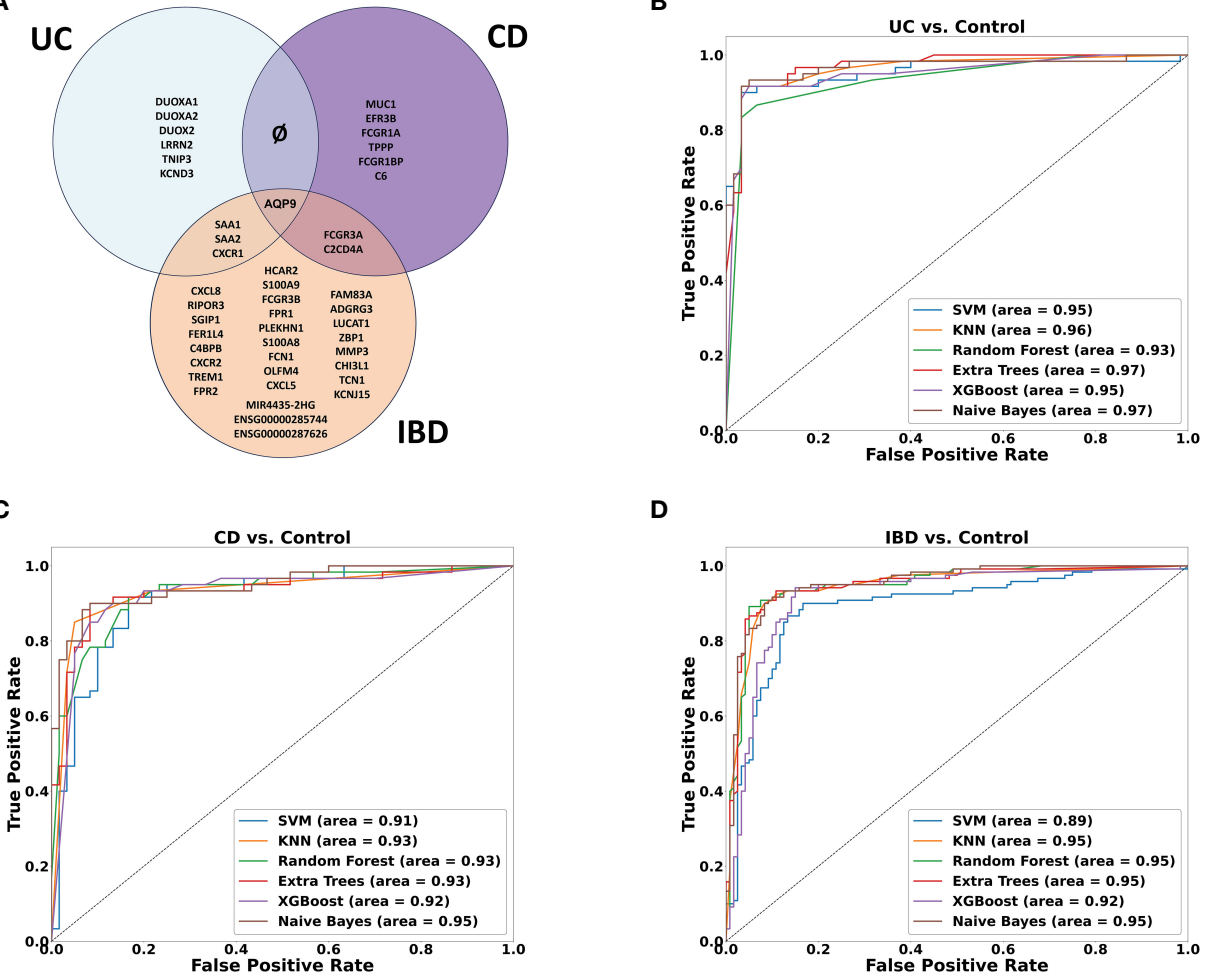
Machine learning results. **(A)** Venn diagram showing the overlap between ML-selected discriminative genes for the following comparisons: UC vs. Control, CD vs. Control and IBD vs. Control. **(B–D)** ROC curve of True positive rate vs. False positive rate at different six models on validation data GSE193677 for **(B)** UC **(C)** CD and **(D)** IBD analyses.

A machine learning analysis aimed at distinguishing between UC and CD identified ten genes (**Supplementary Table 2**). To verify the accuracy of the trained model, we employed two approaches. Initially, we utilized independent UC and CD samples collected from the same tissues used in the training set (rectum and ileum, respectively). This yielded an impressive AUC of 0.97 in ROC analysis (see **Supplementary Figure 4A**). However, validation failed when utilizing external data from the rectum, which represents the same tissue for both diseases, resulting in AUC scores hovering around 0.6 (see **Supplementary Figure 4B**). Acknowledging the limitations posed by tissue effects in discriminating between the diseases, we subsequently, developed diagnostic models to identify genes capable of effectively discriminating between inflamed samples of UC, CD and IBD each compared separately against non-IBD controls. Six ML algorithms were employed with hyperparameter tuning (see methods). We found ten genes distinguishing between UC and non-IBD samples, another 9 genes for the comparison between CD and non-IBD samples, and 34 genes for the differentiation between IBD and non-IBD samples (the genes are indicated in **Figure 5A**). The most accurate models were generated for the classification of UC from non-IBD control samples, followed by the IBD classification, and finally CD classification. Specifically, the highest accuracy scores were achieved with the validation data using KNN and Random Forest. In the UC analysis, Random Forest achieved 0.947 accuracy, KNN reached 0.933. For IBD, both models achieved 0.9 accuracy. In CD, KNN had the highest accuracy of 0.9, followed by Naive Bayes at 0.89. ROC curves were generated for all models and analyses on the validation data (**Figures 5B–D**), illustrating their performance in terms of the true positive rate versus the false positive rate. The curves demonstrated good discriminatory ability, with a notable separation between the diseased and non-IBD control samples in all comparisons. Overall, the results indicate that the classification models achieved a robust and reliable performance in distinguishing between different disease groups and non-IBD controls.

### Novel non-coding RNAs in IBD

**Figure 6:**
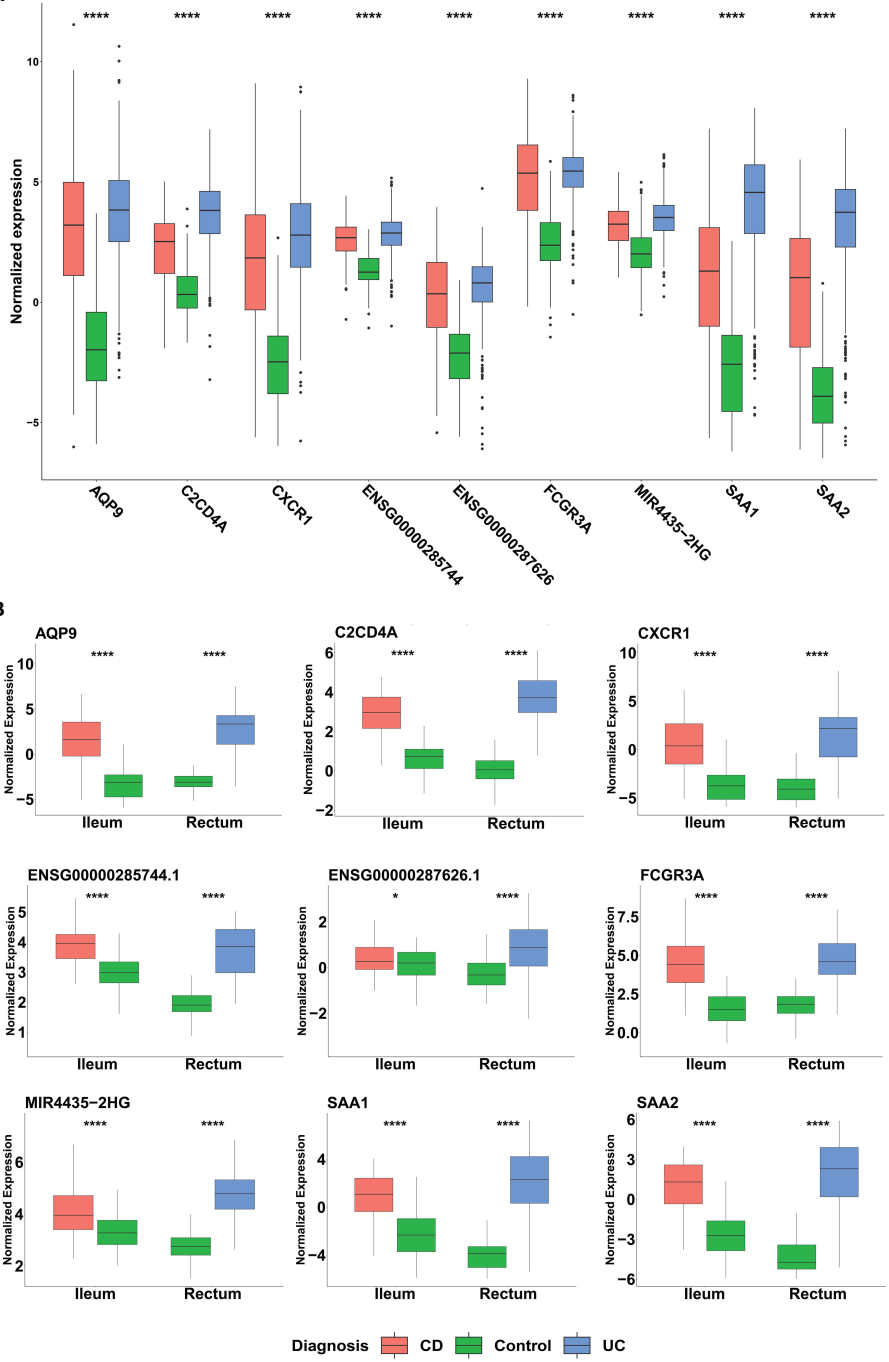
Distribution of the normalized expression of selected genes across UC, CD and non-IBD control samples in **(A)** Mega-analysis dataset: CD (n= 183), UC (n= 386) and control group (n=128). P-values were calculated using two-way analysis of variance (ANOVA). **(B)** GSE193677 validation dataset: The dataset includes inflamed samples from CD (n=60, ileum) and UC (n=60, rectum), along with non-IBD control samples from both the ileum (n=60) and rectum (n=60). Statistical significance was assessed using Student’s t-test. *p < 0.05, ****p < 0.0001 indicate the significance levels.

Among the genes identified through our ML analyses, we recognized known immune related genes and diagnostic markers. Some of the genes were common and intersected between the different models, with *AQP9* present across all of them, *FCGR3A* is present in both CD and IBD analyses, while *SAA1*, *SAA2*, and *KCNDA3* intersect between UC and IBD analyses (**Figure 5A**). Of particular interest, IBD analysis revealed three long non-coding RNA (lncRNA) genes, two are novel transcripts, *ENSG00000285744* and *ENSG00000287626*, along with known lncRNA *MIR4435-2HG*. To verify the differential expression of the six intersected genes along with the lncRNAs, box plots were generated using the normalized data and significant expression level differences were observed in both our mega-analysis dataset and the GSE193677 validation dataset (**Figures 6A, B**), with lower expression of these genes in non-IBD control samples compared to CD and UC. Notably, all genes obtained from the ML analysis showed significant expression levels differences (**Supplementary Figure 5**).

**Figure 7:**
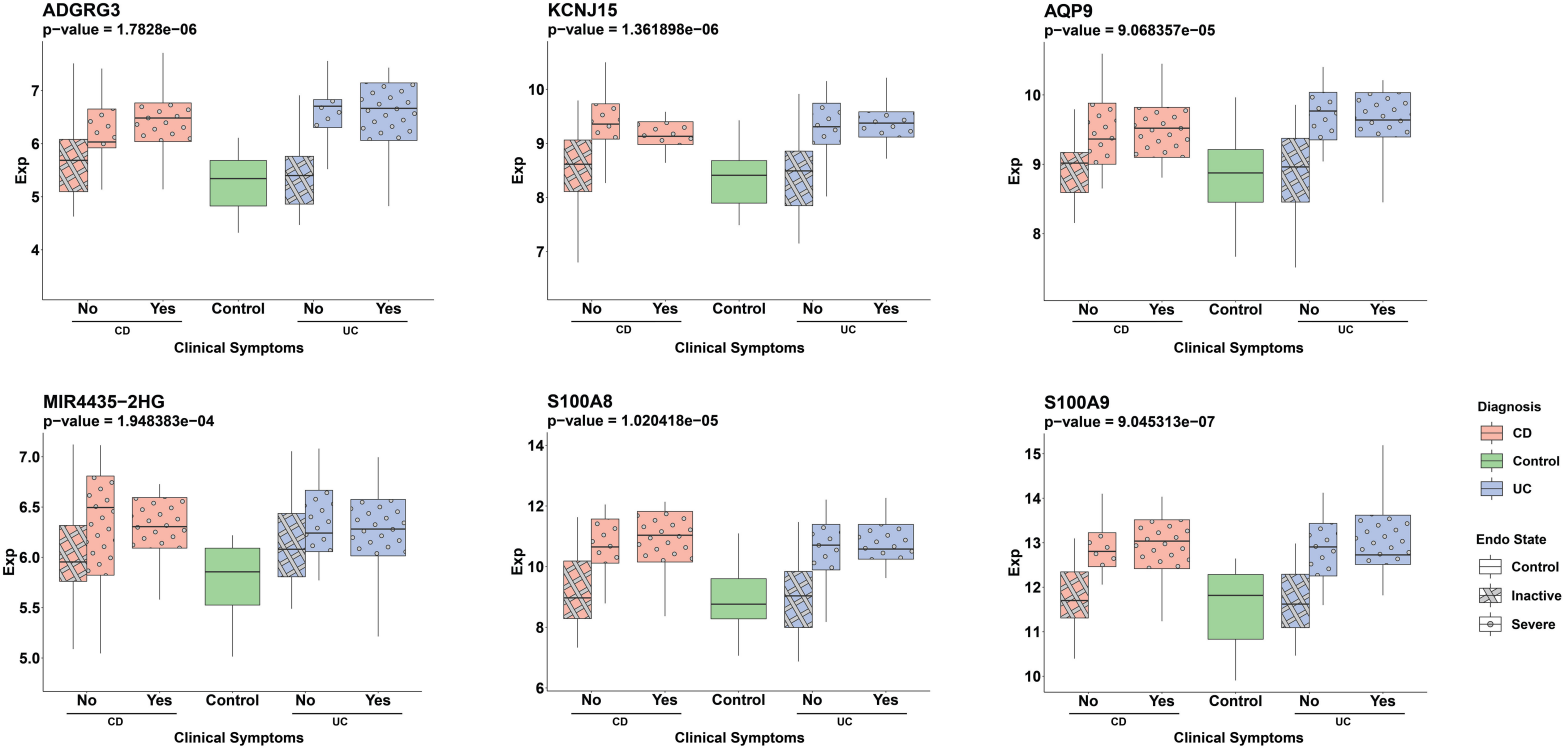
Distribution of ML detected genes’ normalized expression in serum samples across different patient groups. Pink and blue colors represent CD and UC patients, respectively. The pattern differentiation—crosshatch for an inactive endoscopic state and circles for a severe endoscopic state—reflects the severity of endoscopic conditions. The x-axis labels (‘Yes’ or ‘No’) distinguish between the presence or absence of clinical symptoms in patients, with ‘Yes’ indicating clinical symptoms and ‘No’ representing the absence of symptoms. The groups are: CD patients without clinical symptoms and either inactive (*pink, crosshatch*, n=20) or severe (*pink, circle*, n=15) endoscopic states, CD patients with clinical symptoms and severe endoscopic states (*pink, circle*, n=12), UC patients without clinical symptoms and either inactive (*blue, crosshatch*, n=20) or severe (*blue, circle*, n=13) endoscopic states, UC patients with clinical symptoms and severe endoscopic states (*blue, circle*, n=14) and the control group (*green*, n=17). The Wilcoxon test was used to assess statistical significance in the comparison between the severe endoscopic IBD groups and the control group.

To evaluate the potential of the ML-selected genes as non-invasive biomarkers, we assessed their expression in blood samples from IBD patients (**Supplementary Figure 6**). Out of the 34 genes, 29 were available in the blood expression dataset. We observed a significant upregulation of 12 genes (p-value < 0.01) whose expression was higher in individuals with severe endoscopic manifestations of IBD when compared to the control group. The expression of six of these genes, namely *ADGRG3*, *KCNJ15*, *AQP9*, *MIR4435-2HG*, *S100A8*, and *S100A9*, which showed the most significant differences based on their p-values, are depicted in **Figure 7**.

### Connection of drug-gene signature by CMap

Finally, we utilized the Broad Institute Connectivity Map (CMap) (25) to predict potential novel therapeutic compounds based on differential gene expression results. For each the three DEG comparisons described above, we run a separate CMap analysis. In each analysis we used the top 300 DEGs, comprising 150 upregulated and 150 downregulated genes as a query to the CMap system. In addition we run another query that contained the 34 genes selected in IBD ML analysis, 30 of them were recognized by the CMap tool. These four sets of genes were employed as queries in seeking potential connections or similarities in gene expression patterns with drug-induced gene signatures present in the CMap database. It’s worth noting that the CMap system employs different cell lines for its analyses. Given the intestinal nature of our diseases of interest, we placed a specific emphasis on results derived from the HT-29 cell line.

**Figure 8:**
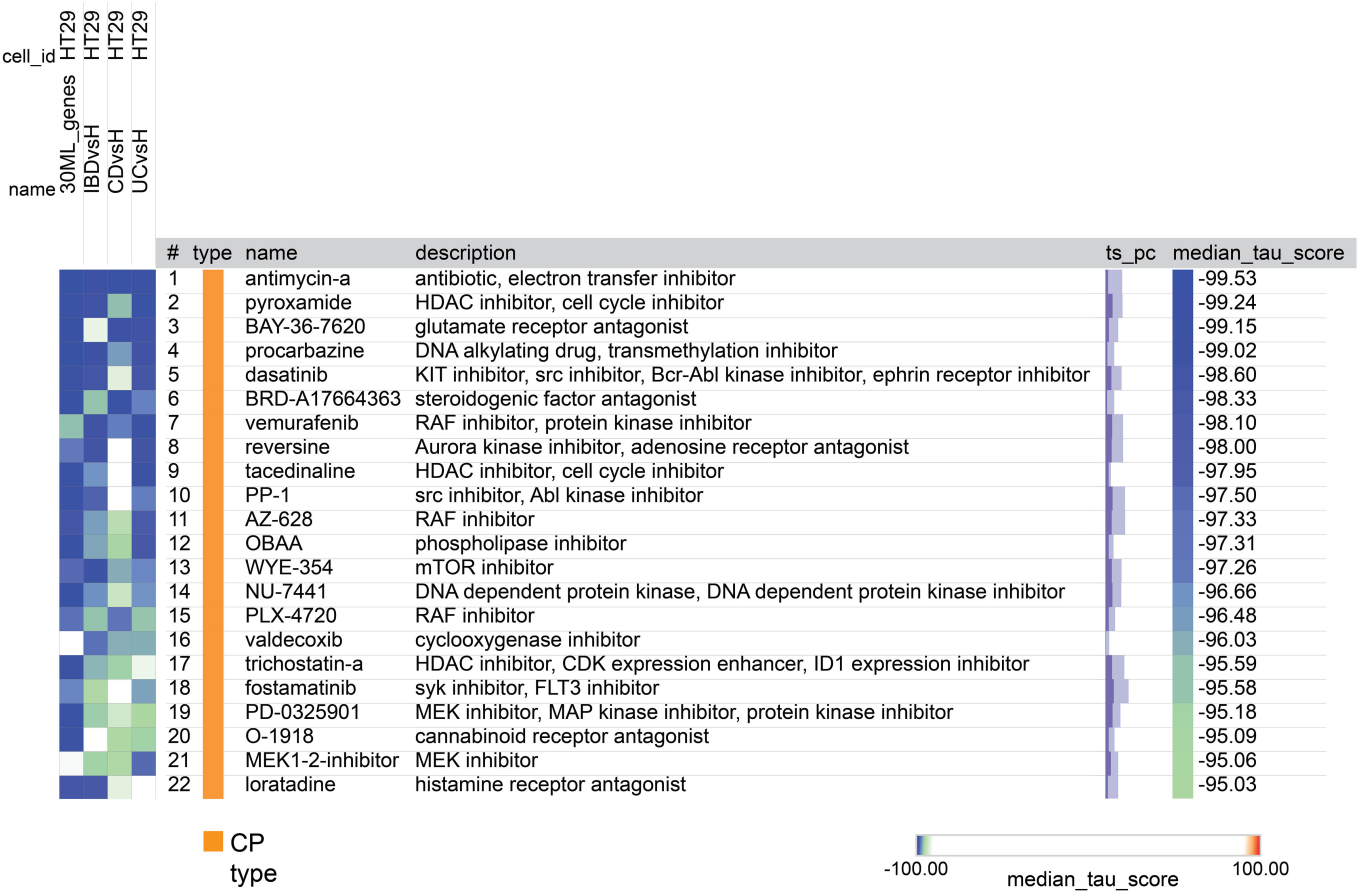
A heatmap generated by CMap showing the connection, assessed by connectivity score (tau score) between gene expression signature of the 4 groups (columns) and the compounds (rows). The score measures the strength of connection between the query signature and a compound. Compounds are sorted by decreasing order of median score across the four groups. Compounds with median score lower than -95 are considered significant.

After the tool analyzed the valid genes, it revealed 22 compounds with median_tau_score values lower than -95. This indicates a strong and significantly opposing biological effect directed towards the gene signatures associated with the various diseases (as depicted in **Figure 8**). The compounds and various families identified in the CMap suggest potential implications for IBD treatment. Notably, Antimycin-A exhibits the highest connectivity score (-99.53), along with Histone deacetylase (HDAC) inhibitors (Pyroxamide, tacedinaline, and trichostatin-a),Monoamine oxidase inhibitor (Procarbazine), BCR-ABL kinase inhibitors (Dasatinib) and RAF inhibitors (Vemurafenib, AZ-628, PLX-4720) mTOR inhibitors (WYE-354) and SYK inhibitors (Fostamatinib) MEK inhibitors (PD-0325901, MEK1-2-inhibitor) Cannabinoid receptor antagonist (O-1918), cyclooxygenase inhibitor (Valdecoxib) and Histamine receptor antagonist (Loratadine).

## Discussion

Whole-transcriptome analyses of IBD samples have been extensively performed over the years, but there is no conclusive understanding regard the mechanisms and expression pattern of specific genes underlying IBD. To gain a deeper insight into the mechanism of IBD, we conducted a mega-analysis, incorporating nearly 700 samples, along with reprocessing over 350 samples of validation data. Utilizing this comprehensive approach, we integrated data from multiple studies and applied unified bioinformatics methods to analyze the combined dataset thoroughly. By doing so, we have demonstrated the expression of genes that are already known to be related to the disease, as well as additional genes and novel transcripts whose expression is different in IBD and can potentially be exploited as a novel therapeutic target. We also characterized different pathways and upstream regulators, which are involved in the disease and can shed light on its underlying mechanism. Finally, using CMap, we presented new potential implications for the treatment of IBD.

In this work, we analyzed both CD and UC collectively as a group of IBD patients and assessed each disease individually. A search in the literature shows that many studies have already identified shared genetic risk factors and common pathways associated with both diseases (37). However, there’s also a growing interest in understanding the distinctions between UC and CD, as these differences can have clinical implications (4). In our attempt to detect genes that can distinguish between CD and UC, we encountered challenges due to tissue-specific effects. MDS analysis of the transcriptomes revealed that region of intestinal biopsy, colon or ileum, had significant effect on gene expression, overshadowing the discriminatory potential of inflammation status, with disease subtype (UC vs CD). We identified 10 genes that effectively distinguished between UC and CD, when testing our training model using samples originated from the same tissue source as used to train the model (UC from rectum and CD from ileum). However, validation using data from external samples obtained from the same tissue (CD and UC from the rectum) resulted in models failure. This aligns with the findings of Argmann et al. who similarly emphasized the dominant influence of the biopsy site on gene expression variation, with minimal divergence observed between UC and CD subtypes (8). To the best of our understanding, many of the differences in gene expression reported in the literature which were attributed to variations between the two diseases, can, in fact, be attributed to differences between the affected tissues rather than the disease themselves, since each of the diseases is mainly expressed in a different segment of the intestine (3). In light of these limitations, our primary focus was on identifying common molecular signatures associated with both UC and CD. We specifically emphasized shared biological pathways and mechanisms, aiming to enhance our understanding of the fundamental processes contributing to IBD. Furthermore, we sought to explore potential therapeutic targets that could be relevant for both diseases, regardless of the specific disease subtype or biopsy location.

Numerous published studies have consistently demonstrated differential gene expression in the inflamed intestinal tissues of individuals with IBD when compared to samples from healthy subjects. For instance, the Dual oxidase (*DUOX*) gene family, responsible for producing reactive oxygen species (ROS) (38) has been a subject of interest in these investigations. *DUOX2* has been linked to very early onset IBD (39), and previous studies have reported overexpression of *DUOX2* and *DUOXA2* genes in UC (40). Our differential gene expression analysis supports these findings, highlighting *DUOX2* and *DUOXA2* among the top ten significantly upregulated genes in samples from IBD patients. Additionally, using ML algorithms, we showed that *SAA1* and *SAA2* discriminate between inflamed samples of UC, as well as IBD and non-IBD controls. This finding has already been published indicating that elevated levels of Serum Amyloid A (SAA) proteins, specifically SAA1 and SAA2, were consistently observed in IBD patients, particularly during active inflammation (41). We also identified *MMP3* (Matrix Metalloproteinase 3), *REG1A* (Regenerating Islet-Derived Protein 1 Alpha), and *CHI3L1* (Chitinase 3-Like 1) among the up-regulated genes in intestine samples of IBD patients. These genes are also known as upregulated in inflamed areas of colons of IBD patients compared to uninflamed tissues (42). Since these genes are involved in tissue remodeling (43–45), their aberrant expression may influence the extracellular matrix and tissue damage observed in IBD inflammation. Finally, of particular interest in our findings are the genes of the channels proteins AQP (Aquaporin) family, namely *AQP9* and *AQP8*. *AQP9* belongs to the aquaglyceroporins subfamily facilitates the membrane transport of water and glycerol and consistently displayed upregulation across UC, CD and IBD patients compared to non-IBD control subjects, demonstrating a commonality that also emerged in all our ML analyses. The exact mechanism of *AQP9* in IBD remains unclear, but its increased expression in IBD has been observed in previous studies (46, 47). Recent research revealed that *AQP9* is downregulated in IBD patients’ responders to infliximab compared to non-responders (48).

In contrast, *AQP8*, a member of the *AQP8*-Type Aquaammoniaporins, is associated with water absorption regulation, particularly in the duodenum, jejunum, and colon. It plays a role in water transport, however distinguishing itself with permeability to NH3/NH4+ (49). This gene exhibited pronounced downregulation across UC, CD and IBD patients compared to non-IBD control subjects. The observed decrease in its expression in IBD patients aligns with previous research findings (50, 51). However, it’s important to note that discovering inhibitors for AQP proteins poses a significant challenge due to their perceived ‘undruggable’ properties (52). Among other down regulated genes, *CLDN8* (Claudin 8) a tight junction protein involved in maintaining the integrity of the intestinal barrier known to be down-regulated in both UC and CD (53). Additionally, *CA1* (Carbonic Anhydrase 1) involved in pH regulation and mucosal protection (54) and other genes involved in transport (*OTOP2* Otopetrin 2, *SLC26A2* (Solute Carrier Family 26 Member 2), in metabolic process *HMGCS2* (3-Hydroxy-3-Methylglutaryl-CoA Synthase 2) and immune regulation (*DPP10* Dipeptidyl Peptidase 10) were also down-regulated. One notable discovery is a novel transcript, *ENSG00000254645*, which is downregulated in IBD patients compared to non-IBD subjects. Recently, this transcript was reported to be down regulated in CD (33). This transcript is a long non-coding RNA (lncRNA) located on chromosome 11, with the *SOX6* (SRY-Box Transcription Factor 6) gene upstream and *INSC* (Inscuteable Spindle Orientation Adaptor) and the calcitonin genes (*CALCa* and *CALCB*) downstream (55–57).

Based on our DEGs analysis we also revealed pathways and upstream regulators associated with bacterial response and the activation of the NFκB transcription factor. These pathways promote the production of pro-inflammatory molecules, including tumor necrosis factor (TNF), interleukins (IL)-1, IL-6, IL-1B, and interferon (IFN-γ) (58). One significant pathway that stood out was the S100 family signaling pathway. It was notably activated in the UC vs. control comparison and ranked among the top ten activated signaling pathways in the CD comparison (data not shown). This pathway plays a crucial role in IBD, particularly through the involvement of S100A8 and S100A9 proteins, collectively known as calprotectin. These proteins are highly expressed by neutrophils and immune cells during inflammation and serve as a fecal calprotectin marker for inflammation and disease activity in UC (59). Another pathway enriched in UC was the IL-17 signaling. This pathway was also ranked in the top 20 pathways in CD and IBD comparisons (data not shown). Elevated IL-17 levels in active IBD patients’ blood and inflamed mucosa underline its role in disease inflammation (60, 61). However, caution is warranted in targeting this pathway for treatment, as observed in clinical studies using IL-17 inhibitors. These trials revealed heightened risks, including increased Candida albicans infections in IBD patients’ intestinal mucosa and severe adverse events leading to the premature cessation of an anti-IL-17 treatment trial for CD (60, 61). Clinical trials targeting the IL17 pathways show improvement in other inflammatory conditions, such as psoriasis, however, their administration has shown links to both new-onset and exacerbation of IBD, emphasizing the complexity and potential risks associated with manipulating this pathway in IBD therapy (62, 63). It’s noteworthy that to date, there are no ongoing clinical trials specifically targeting the IL-17 pathway for inflammatory bowel disease, signifying the need for further research to understand its role and develop safer, more effective treatments tailored to these diseases.

The pathways undergoing downregulation, encompassing metabolic, catabolic, and NR pathways, play a critical role in immune cell functions (64). Among these pathways, the LXR/RXR pathway has role in attenuating proinflammatory responses by modulating NFκB activity (65). Notably, in CD and IBD, we observed downregulation of this pathway alongside activation of the upstream regulator NFκB complex. In UC, while NFκB activation was also evident, the LXR/RXR pathway did not rank among the top 20 downregulated pathways. However, upon closer examination of the downregulated pathways in UC, the LXR/RXR pathway fell just slightly below the significance Z-score cutoff (data not shown). Furthermore, other immunometabolism pathways such as PXR/RXR Activation and the xenobiotic metabolism PXR signaling pathway, which also modulate NFκB activity (66), are downregulated in UC. Notably, NR pathways are significantly influenced by gut microbes (67) and can be a pharmacologically target for autoimmune treatment (34, 68).

Our ML analysis revealed three long non-coding RNAs (lncRNAs) including two novel transcripts, *ENSG00000285744* and *ENSG00000287626*, along with *MIR4435-2HG*. These lncRNAs effectively distinguish between inflamed samples of IBD and non-IBD controls. The *ENSG00000285744* transcript is located on chromosome 8 in proximity to IL-7, a cytokine known to play a role in the inflammatory response (69). The *ENSG00000287626* transcript is located on chromosome 6 and overlaps with *DTNBP1*, a gene associated with the genetic disorder Hermansky–Pudlak syndrome (HPS), which has been reported to affect some patients with IBD (70). Finally, it is worth noting that the lncRNA *MIR4435-2HG* is already known to be associated with the immune system. The suppression of *MIR4435-2HG* has been shown to inhibit macrophage M1 polarization while promoting M2 polarization. This effect contributes to the alleviation of intestinal inflammation in mice with ulcerative colitis through the JAK1/STAT1 signaling pathway (71). Notably, our IPA analysis revealed the activation of both JAK1 and STAT1 upstream regulators in IBD. In addition, *MIR4435-2HG* was found to directly bind to *EZH2* (72), the catalytic subunit of the polycomb repression complex 2 (PRC2). Epithelial *EZH2* serves as an epigenetic determinant in experimental UC by inhibiting TNFα-mediated inflammation and apoptosis (73). In addition, it was shown that *EZH2* suppression ameliorate intestinal inflammation and delay the onset of colitis associated cancer, suggesting the feasibility of *EZH2* inhibitor for the control of IBD (74). Finally, the expression of *MIR4435-2HG* has been found to correlate with the size of intestinal polyps in Colorectal Cancer (CRC) (75) suggesting *MIR4435-2HG* to play a role in colorectal health. Collectively, these observations suggest that *MIR4435-2HG* may serve as a common molecular marker for inflammation-mediated processes and intestinal health.

Given the absence of specific serum markers for the diagnosis and management of IBD, it has become crucial to identify significant differences in serum levels among genes showing substantial expression in mucosal biopsies during severe endoscopic activity, regardless of the state of clinical symptoms. Notably, *ADGRG3, KCNJ15, AQP9, MIR4435-2HG, S100A8*, and *S100A9* have demonstrated such variations. While *S100A8* and *S100A9* calprotectin have exhibited correlations with serum CRP, suggesting systemic inflammation, their utility might be limited to reflect intestinal inflammation (76). *KCNJ15*, identified as a common marker for UC and ankylosing spondylitis, shows promise as a potential diagnostic marker (77). Given the roles of all genes mentioned in the immune system, further validation is essential for their specific implications in IBD.

We performed data-driven analysis based on CMap for identifying new candidate compounds for IBD therapy. As the cell lines used to create the CMap reference database did not include UC or CD cells, we used HT-29 cells, the only intestinal cells in this database. We identified several potential candidate compounds from thousands of options for IBD therapy. Among them, one standout candidate is Antimycin-A boasting the highest connectivity score. This compound, an ATP synthase inhibitor, is recognized for its influence on mitochondrial dysfunction, a hallmark associated with IBD pathogenesis (78). Other compounds identified are Histone deacetylase (HDAC) inhibitors, like Pyroxamide, tacedinaline and trichostatin-a, as potential IBD treatment options. Notably, these compounds are known for their anti-inflammatory properties, offering promise in regulating inflammation linked to IBD (79). Likewise, the Monoamine oxidase inhibitor Procarbazine may have the potential to modulate neurotransmitters and immune responses and BCR-ABL kinase inhibitors such as Dasatinib and RAF inhibitors like Vemurafenib, AZ-628, and PLX-4720 might indirectly impact immune pathways, offering insights into IBD modulation (80, 81). Additionally, mTOR inhibitors, represented by WYE-354, and SYK inhibitors like Fostamatinib are under investigation for their anti-inflammatory effects (82, 83) and MEK inhibitors, including PD-0325901 and MEK1-2-inhibitor, target pathways associated with inflammation and immune responses (84). It’s worth acknowledging the dichotomous findings regarding the impact on IBD as certain reports suggested RAF/MEK inhibitors to potentially promote IBD in humans and mice (85, 86), while others have indicated that MEK inhibitors hold promise in improving diarrhea and histological scores in a murine colitis model (84), and RAF inhibition has induced clinical remission in CD patients (87). Lastly, compounds such as the Cannabinoid receptor antagonist O-1918, cyclooxygenase inhibitor Valdecoxib, and Histamine receptor antagonist Loratadine are all associated with anti-inflammatory pathways (88–90). These findings open new treatment strategies for future studies in IBD management.

In conclusion, our observations of an association between IBD and different candidate genes, specifically the novel transcripts *ENSG00000285744*, *ENSG00000287626*, and the *MIR4435-2HG*, needs to be further validated and interrogated functionally. *MIR4435-2HG* shows promise as a diagnostic tool using a simple blood test and may even serve as a therapeutic target. Finally, the identification of new candidate compounds for IBD therapy shed light on potential avenues for IBD treatment and warrant further investigation.

## Data availability statement

The original data used in the study are included in the article. Further inquiries can be directed to the corresponding author.

## Ethics statement

Ethical approval was not required for the study involving humans in accordance with the local legislation and institutional requirements. Written informed consent to participate in this study was not required from the participants or the participants’ legal guardians/next of kin in accordance with the national legislation and the institutional requirements.

## Author contributions

ES: Investigation, Methodology, Formal analysis, Writing – original draft. TZ: Investigation, Methodology, Conceptualization, Supervision, Writing – review & editing. MK: Writing – review & editing, Formal analysis. LS: Formal analysis, Writing – review & editing. GS: Writing – review & editing, Investigation. MS-D: Investigation, Writing – review & editing, Conceptualization, Methodology, Supervision.
