# Supplementary material for: Exploring potential biomarkers and therapeutic targets in inflammatory bowel disease: insights from a mega-analysis approach
Source: Front Immunol. 2024 Mar 6;15:1353402. doi: 10.3389/fimmu.2024.1353402 (PMC10951083; doi:10.3389/fimmu.2024.1353402)
Supplement: Supplementary file 1 [file Presentation_1.pdf]

Supplementary Methods and Material

Exploring potential biomarkers and therapeutic targets in Inflammatory Bowel Disease: insights from a Mega-Analysis approach

Supplementary Methods

For the detection of DEGs by comparing each disease (CD/UC/IBD) to control samples we used means model as described in the following linear equation:
$expression=\beta_1\ Disease + \beta_2\ Control + \gamma_1\ Study_1 + \ldots + \gamma_{n-1}\ Study_{n-1}$

The corresponding design matrix was defined as
design <- model.matrix(~0+group+studyBatch), where group contained disease (CD/UC/IBD) and control samples.

Note, that in these models, disease and control samples were taken from same tissue.

For the detection of DEGs to include in machine learning models discriminating between UC and CD samples, we had two factor model, disease-type (CD/UC/Control) and tissue (ileum/rectum). We merged the disease-type and tissue to a single group factor as follows:
CD_ileum, UC_rectum, Control_ileum, Control_rectum. Our design matrix was defined as mentioned above, and the comparison of interests were defined as:
1. Interaction term: [CD_ileum - Control_ileum] – [UC_rectum - Control_rectum]
2. Tissue effect: [Control_ileum - Control_rectum]
Only genes having significant interaction, and non-significant tissue effect were included in ML models.

Supplementary Material

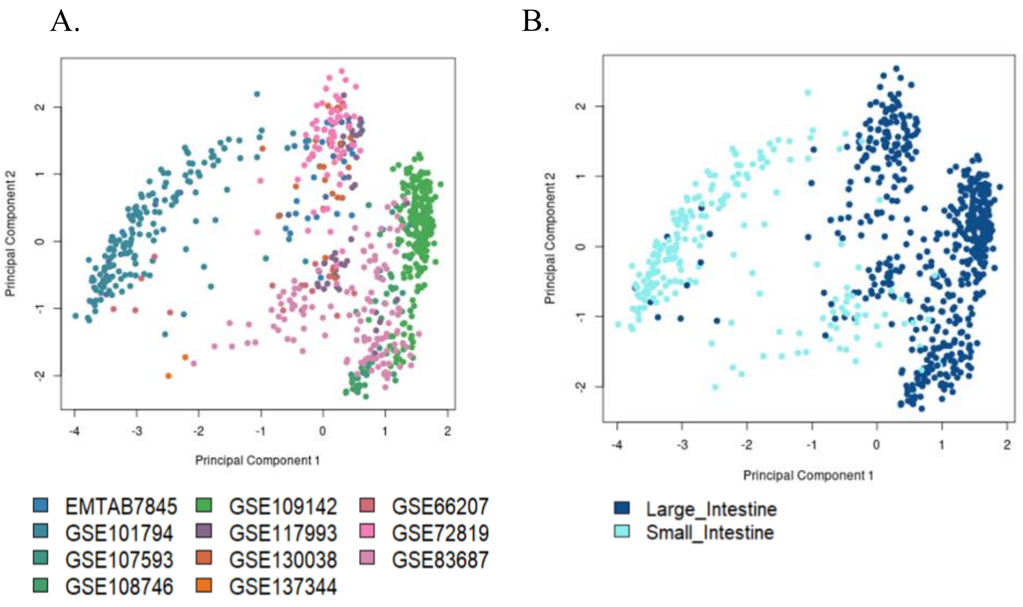

Supplementary Figure 1:
Multidimensional scaling (MDS) plot. (A) Separation of data based on specific study dataset. (B) Separation of data based on Tissue source.

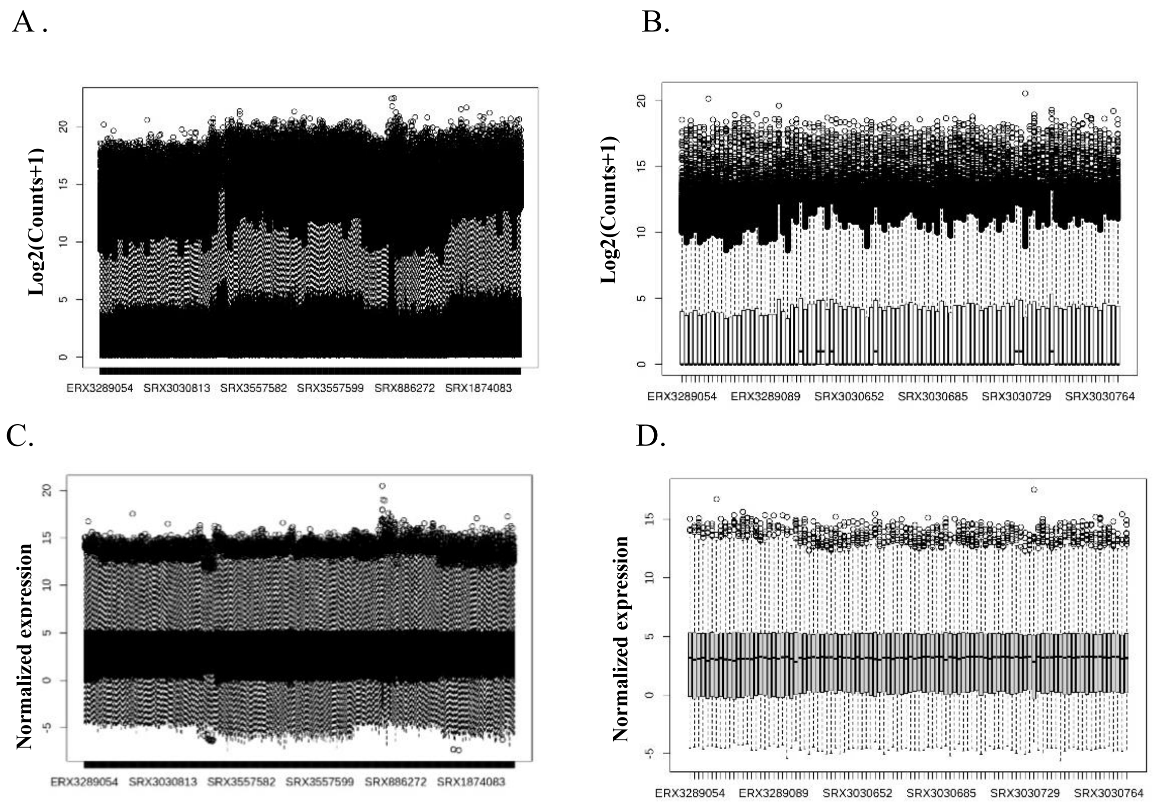

E.

	Mean	Median	SD	Variance	Q1	Q3	Min	Max	IQR
**Raw counts**	345.6878	0	5577.577	31109365	0	30	0	5775495	30
**Normalized counts**	2.075981	2.466036	3.556127	13	-1	5	-7	20	6

Supplementary Figure 2: Boxplots depict the distribution of read counts in each sample before (A-B) and after normalization (C-D). (A) Log2-transformed raw expression counts. (B) Distribution of log2-transformed raw expression counts across 100 random samples. (C) Normalized and voom-transformed counts. (D) Normalized and voom-transformed counts across 100 random samples. (E) Statistics of expression counts data pre- and post-normalization.

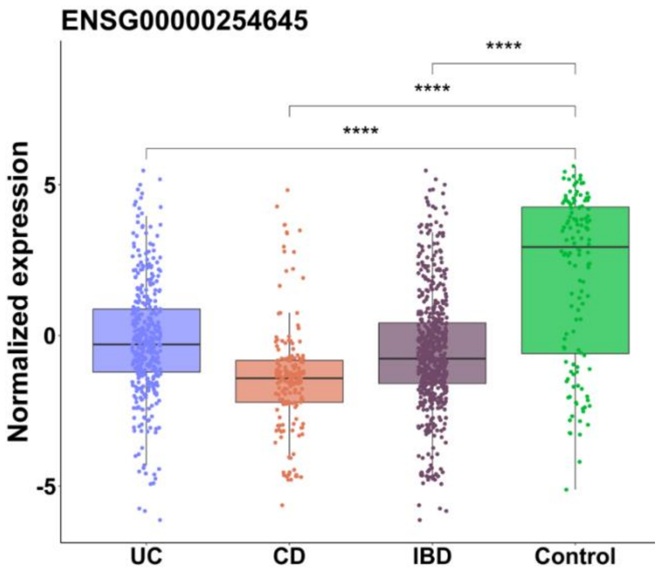

Supplementary Figure 3: Normalized expression of lncRNA ENSG00000254645 across all the different diagnosis. Statistical significance between each diagnosis and the Control group was assessed using Wilcoxon test. ****p < 0.0001 indicate the significance levels.

Supplementary Table 1: The tuned hyperparameters for each model.

	UC vs. Control	CD vs. Control	IBD vs. Control
**SelectKBest**			
K	10	9	34
randomstate	123	456	100
**SVM**			
C	10	1000	100
gamma	0.01	0.001	0.001
kernel	sigmoid	sigmoid	rbf
**KNN**			
n	11	3	17
p	1	2	5
**Random Forest**			
bootstrap	TRUE	TRUE	TRUE
max depth	5	5	10
max features	auto	log2	log2
n estimators	5	6	13
random state	6	80	6061
**Extra Tree**			
bootstrap	TRUE	TRUE	TRUE
max depth	5	5	10
max features	auto	auto	auto
n estimators	10	12	60
random state	6	90	70
**XGBoost**			
learning rate	0.1	0.1	0.8
min child weight	1	3	3
gamma	0	0.7	0.8
n estimators	100	100	300
**Naïve Bayes**			
var smoothing	1	1	1

Supplementary Table 2: Ten genes were selected using selectKbest feature selection method to discriminate between UC and CD.

No.	**Ensembl**	**Symbol**
1	ENSG00000162878.13	PKDCC
2	ENSG00000157005.4	SST
3	ENSG00000173221.14	GLRX
4	ENSG00000197093.11	GAL3ST4
5	ENSG00000196511.14	TPK1
6	ENSG00000076351.13	SLC46A1
7	ENSG00000057149.16	SERPINB3
8	ENSG00000104870.13	FCGRT
9	ENSG00000088367.23	EPB41L1
10	ENSG00000158813.18	EDA

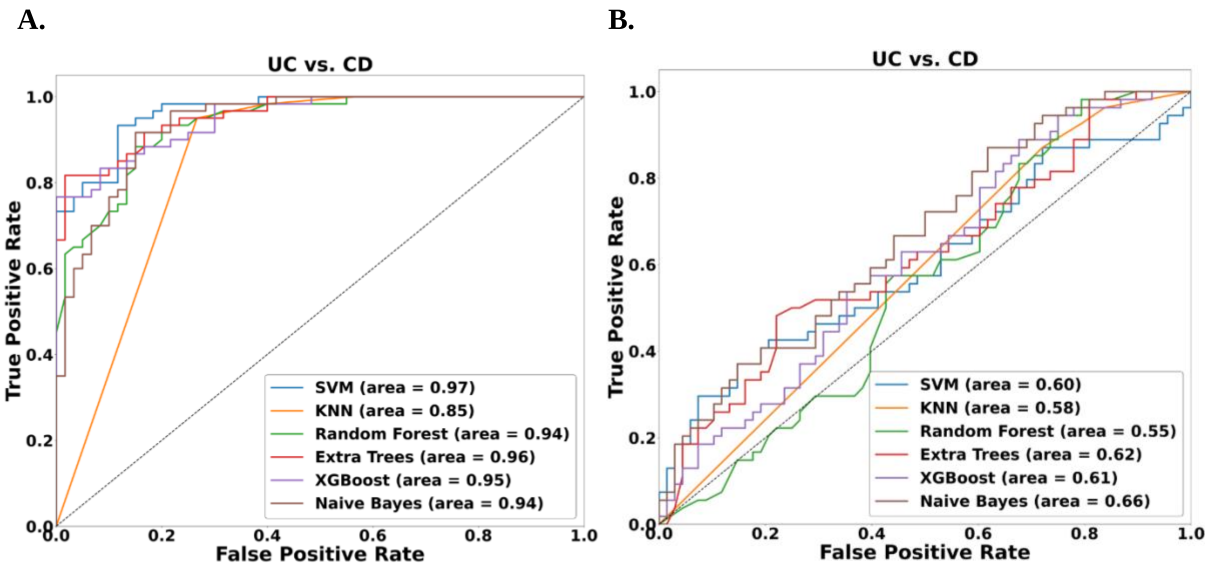

Supplementary Figure 4: ROC curves of True positive rate vs. False positive rate at different six models examined on validation data GSE193677 in UC vs. CD comparison. (A) ROC curve derived from validation set included 60 inflamed samples of UCfrom the rectum and 60 inflamed samples of CD from the ileum. (B) ROC curve derived from validation set, samples included in the analysis originated from the same inflamed tissue in both diseases, UC rectum (n = 54) and CD rectum(n = 68).

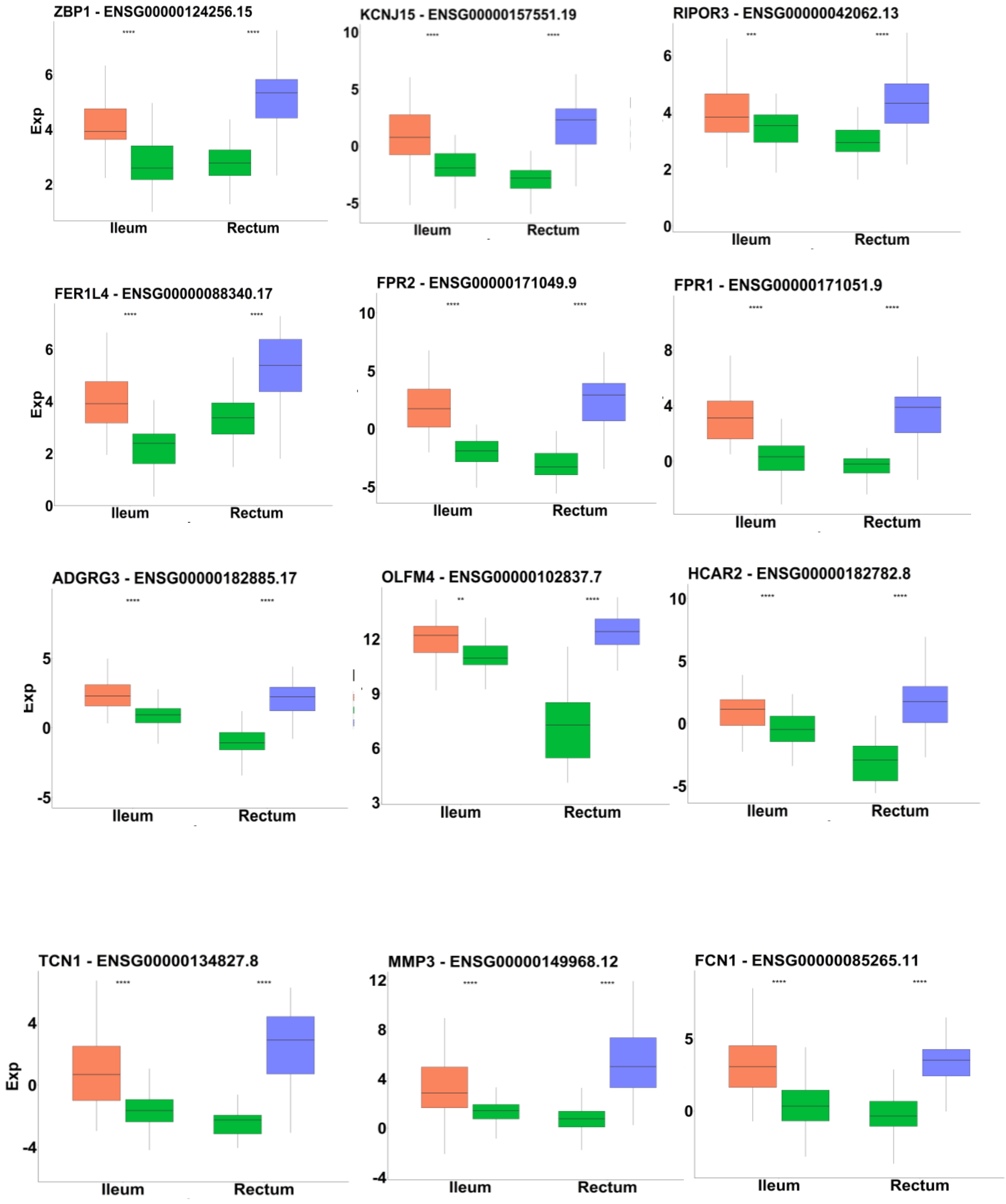
ZBP1 - ENSG00000124256.15
Exp
6
4
2
Ileum
Rectum
KCNJ15 - ENSG00000157551.19
10
5
0
-5
Ileum
Rectum
RIPOR3 - ENSG00000042062.13
6
4
2
0
Ileum
Rectum
FER1L4 - ENSG00000088340.17
Exp
6
4
2
0
Ileum
Rectum
FPR2 - ENSG00000171049.9
10
5
0
-5
Ileum
Rectum
FPR1 - ENSG00000171051.9
8
4
0
Ileum
Rectum
ADGRG3 - ENSG00000182885.17
Exp
5
0
-5
Ileum
Rectum
OLFM4 - ENSG00000102837.7
12
9
6
3
Ileum
Rectum
HCAR2 - ENSG00000182782.8
10
5
0
-5
Ileum
Rectum
TCN1 - ENSG00000134827.8
Exp
4
0
-4
Ileum
Rectum
MMP3 - ENSG00000149968.12
12
8
4
0
-4
Ileum
Rectum
FCN1 - ENSG00000085265.11
5
0
Ileum
Rectum

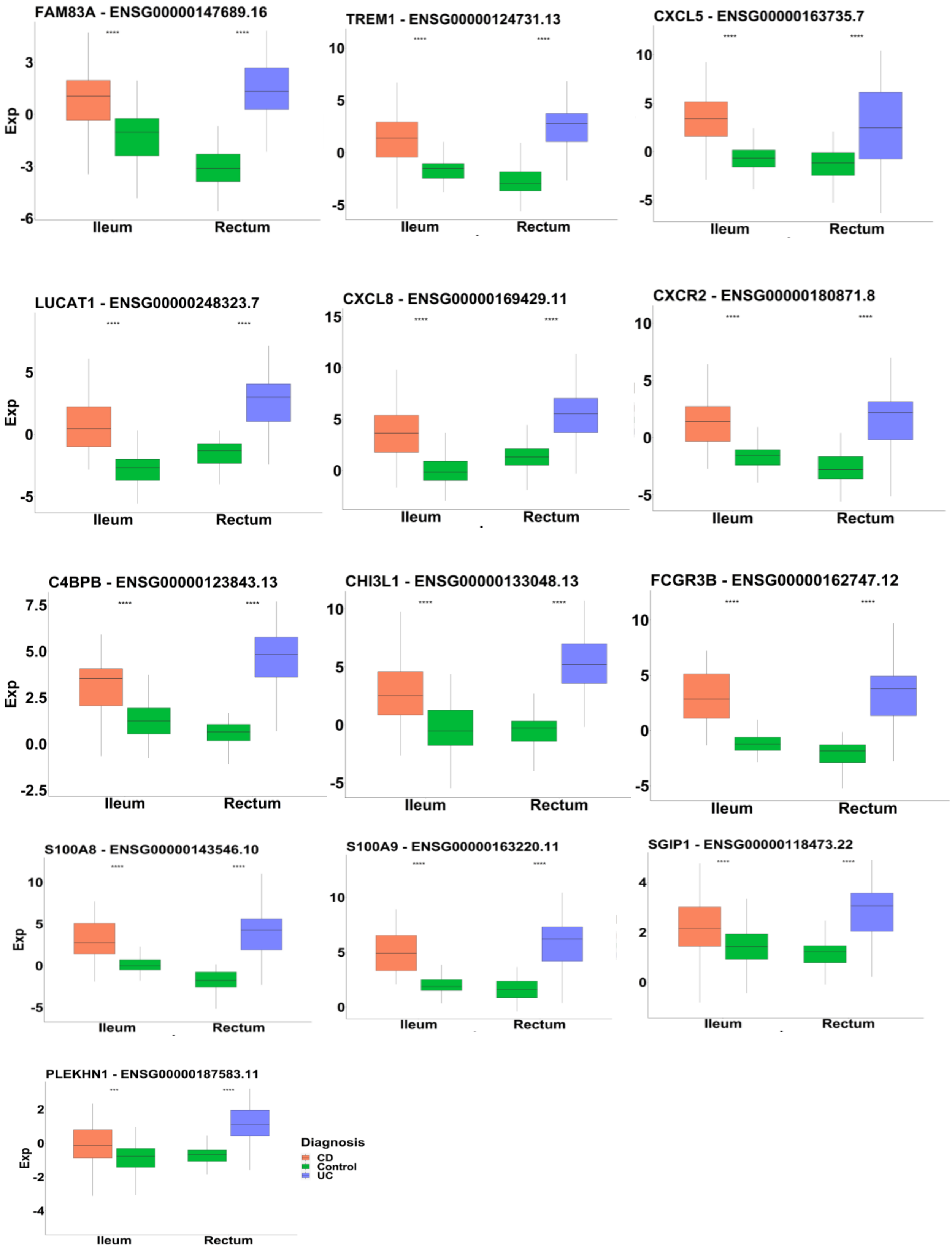

Supplementary Figure 5: Expression of selected genes across UC, CD and Control samples in GSE193677 validation dataset: The dataset includes inflamed samples from CD (n=60, ileum) and UC (n=60, rectum), along with control samples from both the ileum (n=60) and rectum (n=60). Statistical significance was assessed using Student's t-test. **p < 0.01, ***p < 0.001, and ****p<0.0001 indicate the significance levels.

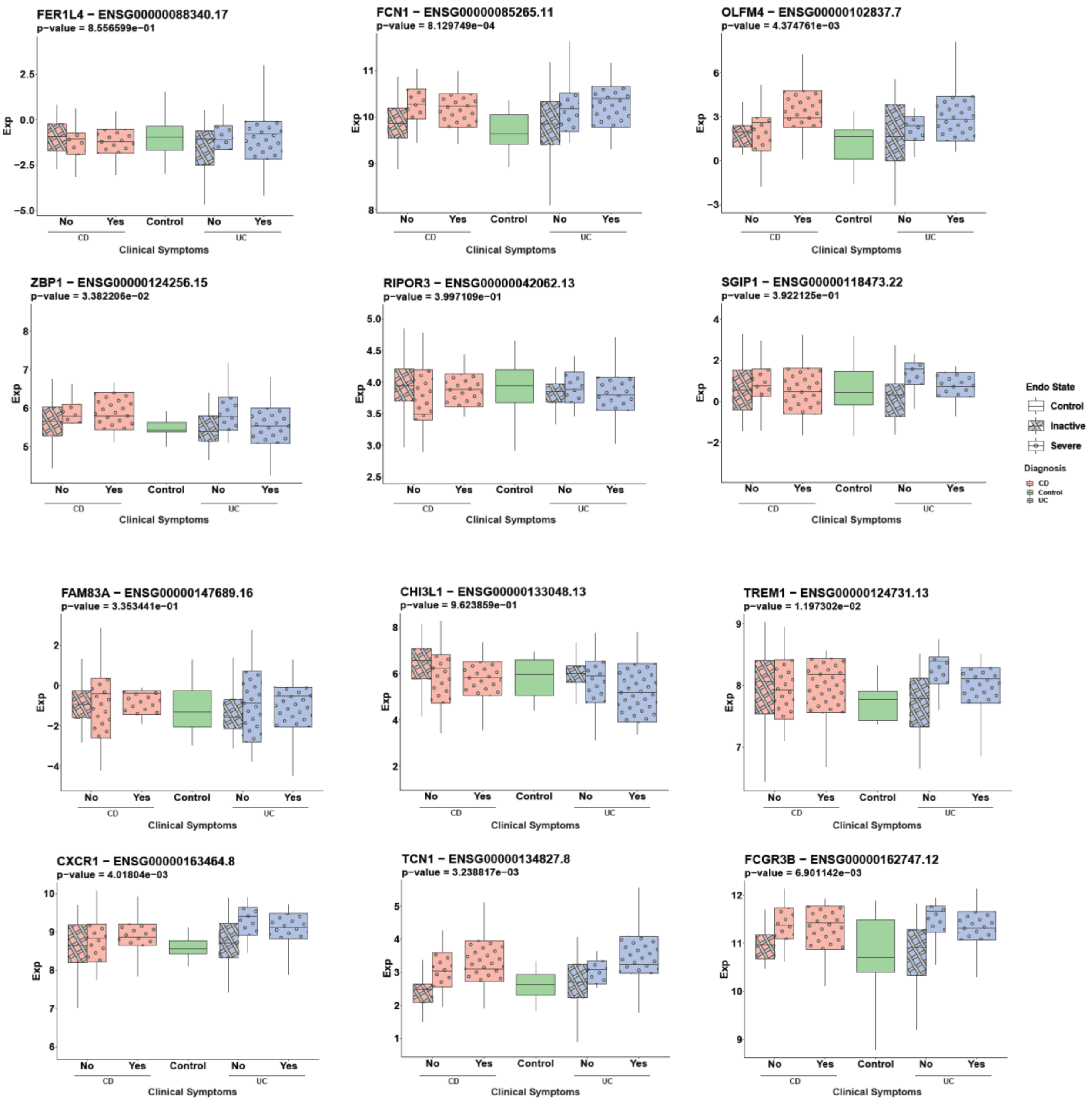
FER1L4 − ENSG00000088340.17
p−value = 8.556599e−01
FCN1 − ENSG00000085265.11
p−value = 8.129749e−04
OLFM4 − ENSG00000102837.7
p−value = 4.374761e−03
ZBP1 − ENSG00000124256.15
p−value = 3.382206e−02
RIPOR3 − ENSG00000042062.13
p−value = 3.997109e−01
SGIP1 − ENSG00000118473.22
p−value = 3.922125e−01
FAM83A − ENSG00000147689.16
p−value = 3.353441e−01
CHI3L1 − ENSG00000133048.13
p−value = 9.623859e−01
TREM1 − ENSG00000124731.13
p−value = 1.197302e−02
CXCR1 − ENSG00000163464.8
p−value = 4.01804e−03
TCN1 − ENSG00000134827.8
p−value = 3.238817e−03
FCGR3B − ENSG00000162747.12
p−value = 6.901142e−03
Exp
No
Yes
Control
CD
UC
Clinical Symptoms
Endo State
Control
Inactive
Severe
Diagnosis
CD
Control
UC

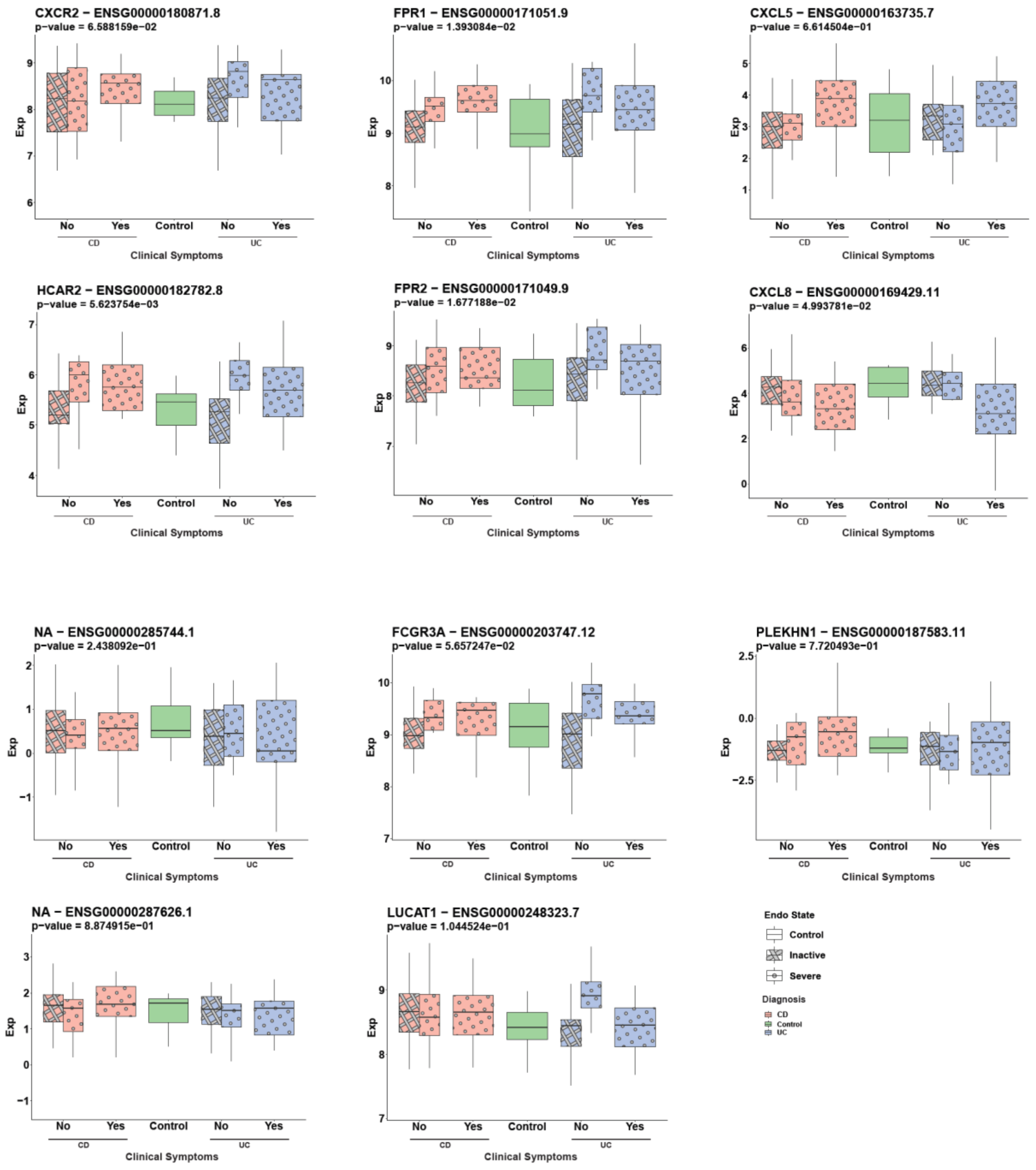

Supplementary Figure 6: Distribution of ML detected genes' normalized expression in serum samples across different patient groups. Pink and blue colors represent CD and UC patients, respectively. The pattern differentiation—crosshatch for an inactive endoscopic state and circles for a severe endoscopic state—reflects the severity of endoscopic conditions. The x-axis labels ('Yes' or 'No') distinguish between the presence or absence of clinical symptoms in patients, with 'Yes' indicating clinical symptoms and 'No' representing the absence of symptoms. The groups are: CD patients without clinical symptoms and either inactive (*pink, crosshatch,* n=20) or severe (*pink, circle,* n=15) endoscopic states, CD patients with clinical symptoms and severe endoscopic states (*pink, circle*, n=12), UC patients without clinical symptoms and either inactive (*blue, crosshatch*, n=20) or severe (*blue, circle,* n=13) endoscopic states, UC patients with clinical symptoms and severe endoscopic states (*blue, circle,* n=14) and the control group (*green*, n=17). The Wilcoxon test was used to assess statistical significance in the comparison between the severe endoscopic IBD groups and the control group.
